# Automated longitudinal treatment response assessment of brain tumors: A systematic review

**DOI:** 10.1093/neuonc/noaf037

**Published:** 2025-02-12

**Authors:** Tangqi Shi, Aaron Kujawa, Christian Linares, Tom Vercauteren, Thomas C Booth

**Affiliations:** School of Biomedical Engineering & Imaging Sciences, King’s College London, London, UK; School of Biomedical Engineering & Imaging Sciences, King’s College London, London, UK; School of Biomedical Engineering & Imaging Sciences, King’s College London, London, UK; School of Biomedical Engineering & Imaging Sciences, King’s College London, London, UK; Department of Neuroradiology, Ruskin Wing, King’s College Hospital NHS Foundation Trust, London, UK

**Keywords:** brain tumor, machine learning, treatment response

## Abstract

**Background:**

Longitudinal assessment of tumor burden using imaging helps to determine whether there has been a response to treatment both in trial and real-world settings. From a patient and clinical trial perspective alike, the time to develop disease progression, or progression-free survival, is an important endpoint. However, manual longitudinal response assessment is time-consuming and subject to interobserver variability. Automated response assessment techniques based on machine learning (ML) promise to enhance accuracy and reduce reliance on manual measurement. This paper evaluates the quality and performance accuracy of recently published studies.

**Methods:**

Following PRISMA guidelines and the CLAIM checklist, we searched PUBMED, EMBASE, and Web of Science for articles (January 2010–November 2024). Our PROSPERO-registered study (CRD42024496126) focused on adult brain tumor automated treatment response assessment studies using ML methodologies. We determined the extent of development and validation of the tools and employed QUADAS-2 for study appraisal.

**Results:**

Twenty (including 17 retrospective and 3 prospective) studies were included. Data extracted included information on the dataset, automated response assessment including pertinent steps within the pipeline (index tests), and reference standards. Only limited conclusions are appropriate given the high bias risk and applicability concerns (particularly regarding reference standards and patient selection), and the low-level evidence. There was insufficient homogenous data for meta-analysis.

**Conclusions:**

The study highlights the potential of ML to improve brain tumor longitudinal treatment response assessment. Interpretation is limited due to study bias and limited evidence of generalizability. Prospective studies with external datasets validating the latest neuro-oncology criteria are now required.

**Key Points**

1. The systematic review emphasizes the role of machine learning in enhancing precision and efficiency in neuro-oncology longitudinal assessments.
2. The review highlights the necessity for further research to address biases and enhance clinical applicability.

**Importance of the Study**

We present the first systematic review that evaluates machine learning (ML) applications for the longitudinal treatment response assessment of brain tumors. Such technologies have the potential to improve neuro-oncological practice, offering a more precise, consistent, and efficient approach to treatment monitoring in both the clinic and during trials. We highlight the need for addressing bias risks in the development of automated ML methods. Despite the potential of ML to improve segmentation accuracy and efficiency, systematic errors appear to be common when the enhancing tumor region is measured. From this published work, automated tools do not appear clinic-ready, and further research, especially incorporating external test datasets and prospective datasets, is now needed for more robust validation. Successful demonstration of tool use in the clinic or in clinical trials is also now required to complete clinical validation.

Brain tumors present significant clinical challenges. For example, due to their infiltrative nature, diffuse gliomas typically have a very poor prognosis with the most common type, glioblastoma, having a median overall survival of only 14.6 months despite standard-of-care treatment (which typically consists of maximal safe tumor resection, followed by radiotherapy with concomitant and adjuvant temozolomide chemotherapy).^1^ The 2-year survival rate is around 30%. Similarly, the presence of brain metastases, which occur in approximately 10% to 20% of adult cancer patients,^2^ also represents a challenging clinical scenario due to the blood–brain barrier influencing systemic therapeutic delivery. Metastatic invasion therefore complicates treatment decisions and is often associated with a median survival of just a few months. For example, patients with multiple brain metastases treated with whole-brain radiotherapy alone have a median survival of about 3–6 months.^3^ To help navigate brain tumor patient management after the initiation of treatment, response assessment using longitudinal imaging has become the clinical standard of care. Regularly scheduled imaging facilitates tracking tumor biology and assessing treatment efficacy, which are important factors influencing decision-making during multidisciplinary team meetings (MDTM or Tumor Boards). The rationale is that disease progression may be identified before clinical symptoms emerge and that may lead to an early intervention—which may plausibly improve therapeutic outcomes and prevent irreversible complications.^4,5^

Longitudinal imaging forms the basis of reference standards for response assessment in clinical trials. In such a research setting, RANO (Response Assessment in Neuro-Oncology) criteria,^6–9^ have become essential by providing a standardized approach for assessing the effectiveness of treatments for brain tumors (Box 1). It is important to note that the US Food and Drug Administration (FDA) has endorsed treatment outcomes based on RANO criteria,^10^ which ensures that they meet the rigorous standards necessary for regulatory approval in clinical trials. The RANO criteria not only consider changes in tumor size and morphology but also include the patient’s clinical presentation and neurological functional status. Standardized clinico-radiological response assessment criteria not only allow comparison of outcomes during trials but also during routine clinical assessment when applied in an expedient and simplified form to help clinicians quickly make reliable treatment decisions given the complexities of interpreting MRI data.^11^

Box 1: **Key Terminology Relevant for Assessing Autonomous Treatment Response Assessment Studies**

Reference Standard: The “reference standard” refers to the best available method to determine the accuracy of diagnostic assessments, establishing a benchmark for evaluating new methods.^12^ Here, radiologists’ (1) manual image segmentation and (2) manual tumor assessment—using expert measurement of the Response Assessment in Neuro-Oncology (RANO) criteria—serve as the reference standards.

Response Assessment in Neuro-Oncology (RANO) criteria: The RANO criteria serve as a standardized set of guidelines for evaluating the effectiveness of brain tumor treatments in clinical trials. These criteria were developed to address limitations in previous assessment methods such as the MacDonald criteria.^13^ RANO assessment focuses on changes in tumor size (typically using the product of bidimensional perpendicular diameters), measured by T1-weighted post-contrast and T2/FLAIR MRI sequences. RANO assessment also incorporates clinical factors (eg, corticosteroid use and neurological symptoms) alongside imaging. Beyond clinical trials, assessments in routine clinical practice may also use RANO or may largely be based on RANO criteria.^11^ First designed for high-grade glioma,^6^ updates and extensions of the RANO criteria have been proposed including for specific tumor types (eg, low-grade gliomas, metastases, meningiomas) and advanced therapies (eg, immunotherapies). Tumor response can be categorized as progression, stable disease, partial response, or complete response, and the criteria are defined.

Index test: The “index test” refers to the new diagnostic test or assessment method under investigation,^12^ which in this review is the automated ML-based assessment, encompassing both segmentation and tumor response evaluation.

Manual longitudinal assessments based on structural MRI protocols can be problematic due to several factors. High-grade gliomas, for example, exhibit a variety of shapes, and their boundaries can be difficult to precisely define. Moreover, the solid tumor often manifests as a cavity rim, making it challenging to capture the full extent accurately. Indeed, in some cases, large cyst-like high-grade gliomas may not meet the “measurable” criteria unless a solid peripheral nodular component of sufficient size (≥10 mm) is present. These complexities highlight the limitations of manual assessments, as they rely on subjective interpretation and can result in inaccuracies in tumor measurement and monitoring, as well as the need for more standardized and objective approaches. Manual assessments are also resource-intensive. In response to these challenges posed by manual assessments in structural MRI protocols, there has been a notable advancement in the development of automated assessment tools. These tools, utilizing various machine learning (ML) methodologies (Box 2),^14,15^ aim to automate—and optimize—the longitudinal assessment of treatment response in brain gliomas. In particular, these automated systems are designed to address the limitations of manual assessments by offering more accurate, reproducible, and efficient methods for evaluating treatment responses and tumor metrics. In this systematic review, we aimed to analyze and summarize the diagnostic accuracy of current ML algorithms used for longitudinal treatment response assessment. While our primary objective was to examine the overall automated treatment response assessment based on ML, a secondary objective was to investigate the underlying automated tumor segmentation.

Box 2: **Overview of Methods in Artificial Intelligence**

Artificial Intelligence (AI) encompasses a wide array of computational techniques aimed at enabling machines to mimic human intelligence. Within AI, machine learning (ML) represents a subset of algorithms that learn complex patterns from data without explicit programming for each specific outcome, to produce analytical models that can make predictions. Neural networks are a key ML approach inspired by the human brain’s structure and use interconnected nodes (like neurons) to process data through sequential layers which perform the pattern recognition process. Deep learning (DL), a subset of ML, uses neural network architectures with multiple layers such as convolutional neural networks (CNNs). CNN architectures like U-Net can be used for specialized tasks like image segmentation. In neuro-oncology, CNN models can be applied to MRI scans to segment tumors or to produce diagnostic, prognostic, predictive, or monitoring biomarkers.^16^ Other deep learning examples in neuro-oncology might use even more advanced neural network architectures like generative adversarial networks (GANs) that can be used to synthesize specific MRI sequences, such as generating contrast-enhanced T1-weighted images of tumors. In summary, AI techniques can be widely applied to clinical decision-making (using a variety of data including images) and image analysis which can enhance the efficiency and accuracy of tasks such as tumor segmentation, treatment response assessment, and disease prognosis.

## Methods

This systematic review was registered with PROSPERO (CRD42024496126). The review was organized in line with the Preferred Reporting Items for Systematic Reviews and Meta-Analysis (PRISMA).^17^ Where appropriate, both Quality Assessment of Diagnostic Accuracy Studies 2 (QUADAS-2) methodology^18^ alongside ML metrics from the Checklist for Artificial Intelligence in Medical Imaging (CLAIM) were used to assess the risk of bias for each study.^19^

### Search Strategy and Selection Criteria

**Figure 1.**
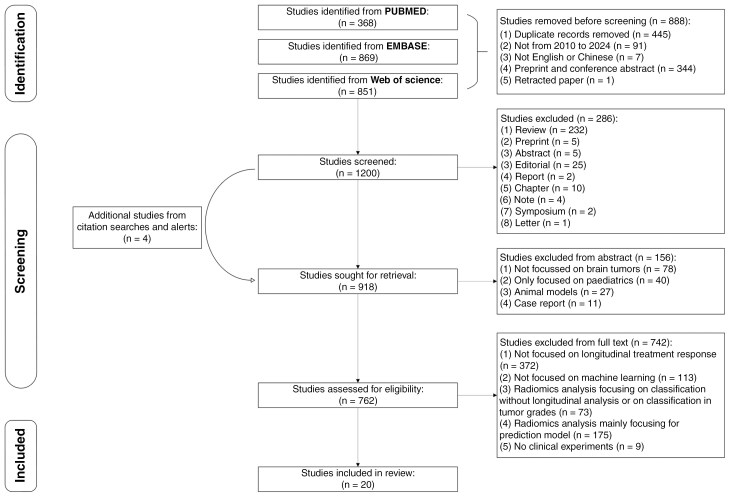
Flow diagram of search strategy. The flowchart depicts the systematic review search strategy and selection process. Initially, 2088 records were identified from PUBMED, EMBASE, and Web of Science databases of which 1200 records were examined further. Of these, 918 studies were retrieved for detailed evaluation, including 4 additional studies extracted from citation searches. Following abstract analysis, 762 full-text articles were assessed for eligibility. This process culminated in the inclusion of 20 studies in the final analysis.

Search terms were applied to PUBMED, EMBASE, and Web of Science databases to extract original research articles published from January 2010 to November 2024 encompassing both text words and database-specific subject headings (Supplementary Table S1). Specifically, we used Medical Subject Headings (MeSH) for PUBMED; EMTREE subject heading terms for EMBASE; and a broader combination of keywords for Web of Science. For example, our PUBMED search strategy was as follows: we used the keywords combination (“automated” or “automatic” or “pipeline” or “AI” or “artificial intelligence” or “ML” or “machine learning” or “deep learning” or “radiomics”) AND (“brain tumor” or “brain metastases” or “glioma” or “glioblastoma”) AND (“longitudinal” or “follow-up” or “follow up” or “treatment response” or “monitoring biomarker” or “response assessment” or “monitoring”) AND (“MRI” or “magnetic resonance imaging” or “magnetic resonance” or “MR”), including both English and Chinese language publications, with a date range from January 1, 2010, to November 25, 2024. The 2010 starting point was chosen because it coincided with the publication of the first RANO paper.^6^ Preprints, abstracts, reviews, editorials, reports, letters, book chapters, case reports, symposiums, retracted papers, or articles without peer review were excluded (Figure 1). The references of all selected articles were hand-searched to identify any potentially relevant studies missed in the initial database search.

Studies were deemed eligible for inclusion if they assessed the longitudinal treatment response of any type of brain tumor (ie, both benign and malignant tumors) using ML methodologies. In the context of this study, ML methodologies are those that enable computers to learn from retrospective data, by automatically tuning algorithms that are not solely composed of explicit instructions, and autonomously make decisions or predictions for prospective use. The definition includes deep learning (DL) which is a type of ML based on artificial neural networks in which multiple layers of processing are used to extract progressively higher-level features from data. The patient cohort was restricted to adult patients (≥18 years old) who underwent standardized treatment and subsequent imaging to evaluate treatment outcomes. Ineligible studies included those reporting on only pediatric populations; those without clinical experiments; and those using only animal models. We also excluded all studies without longitudinal analysis, for example prognostic, diagnostic, or monitoring biomarkers using a single timepoint to determine treatment response by radiomic analysis. Research that only compared pre-operative and early post-operative (ie, <72 hours) imaging timepoints cannot be considered as providing longitudinal assessments of treatment response and was thus excluded.

A meta-analysis could not be performed due to a lack of sufficient homogenous studies identified from the systematic review and marked heterogeneity in the methodology of these included studies.

### Data Extraction

A neuroimaging data scientist, T.S., with 2 years of experience in neuroimaging applied to neuro-oncology, independently performed the data extraction and quality assessment. A.K., a neuroimaging data scientist with 8 years of experience in neuroimaging applied to neuro-oncology also independently performed the data extraction. A junior neuro-oncologist (UK specialist trainee grade; US fellow equivalent), C.A.L., with 5 years of experience in neuroimaging applied to neuro-oncology, also independently performed the quality assessment. Discrepancies between the reviewers were considered at research meetings with a senior neuroradiologist (UK consultant; US attending equivalent) T.C.B. with 17 years’ experience in neuroimaging applied to neuro-oncology, and T.V., a neuroimaging data scientist with 15 years of experience in neuroimaging applied to neuro-oncology, until a consensus was reached.

Data extracted from each study included the type and grade of brain tumor, and whether classification was based on WHO 2016 or 2021 criteria^20,21^; whether data was obtained from single or multiple institutions; the size of training and testing sets; the types of MRI sequences used; and whether the study was retrospective or prospective. We also analyzed the automated models for longitudinal assessment (assigned as index tests) and any automated sub-components within the pipeline, as well as the reference standard applied (eg, RANO 2010). Performance metrics for sub-components prior to longitudinal assessment eg, segmentation, were collected alongside longitudinal assessment performance metrics. Performance metrics extracted were based on index test results compared to reference standard results. Depending on the task, metrics extracted included, for example, Dice coefficient, intraclass correlation coefficient (ICC), or area under the receiver operating characteristic curve (AUC) values.

### Risk of Bias Assessment

To evaluate diagnostic accuracy, we applied QUADAS-2 (Quality Assessment of Diagnostic Accuracy Studies 2) methodology,^18^ a tool specifically designed for the systematic assessment of the quality of diagnostic accuracy studies. This analytical framework facilitates the appraisal of the risk of bias and applicability concerns across 4 key domains: patient selection, index tests, reference standards, and flow and timing. A 3-tiered rating system comprising “low,” “high,” or “unclear” risk of bias or applicability concerns, was employed.

Each domain was systematically evaluated with carefully prepared criteria. For patient selection, we appraised key aspects such as whether participants were enrolled in the study consecutively or at random, followed a standard treatment protocol, and were subject to any inappropriate exclusions. We also evaluated studies as to whether all appropriate exclusions had been applied. For the index test and reference standard domains, we appraised whether blinding was employed during respective formulation or evaluation and whether relevant clinical confounding characteristics had been accounted for. We also confirmed whether all participants were included in the analysis and whether the same reference standard had been applied to them to ensure uniformity. We also determined whether pre-specified thresholds prior to index testing had been fixed a priori.

## Results

In all, 2088 citations fulfilled the search criteria of which the full text of 762 potentially eligible articles were reviewed (Figure 1). A total of 20 studies were included in the final analysis and are listed in Table 1^22–41^.

**Table 1.** Studies Applying Machine Learning to Automated Longitudinal Treatment Response Assessment for Brain Tumors

Study	Dataset 1. Tumor type 2. Retrospective/ Prospective 3. Single/Multiple sites 4. Sequences 5. Numbers	Pipeline steps	Automated model(s) (index test(s), in comparison with reference standard(s))	Performance metrics for tasks prior to longitudinal assessment eg, segmentation	Longitudinal performance metrics
Chang, et al.^22^	Dataset 1 Pre-operative cohort: 1. Grade II–IV glioma (pre 2021 WHO classification) 2. Retrospective 3. Multisite (4 sites) 4. T1 C, FLAIR 5. 843 patients with 843 MRIs (3 site cohorts split 4:1 into train/test sets, one site as external test set with 157 patients) Dataset 2 Post-operative cohort: 1. Grade IV glioma (Glioblastoma) (pre 2021 WHO classification) 2. Retrospective 3. Single-site 4. T1, T1 C, FLAIR 5. 54 patients with 713 MRIs (train: test = 4: 1) Dataset 3: A randomly selected cohort from Dataset 1 and 2: 42 patients (30 train; 12 test)	1. Preprocessing a. Resampling b. N4 bias correction c. Registration d. Intensity normalization e. Skull-stripping (Brain extraction) 2. Segmentation 3. Modified RANO^8^-based bi-dimensional product calculation	3D U-Net compared to expert manual extraction and segmentation Automated modified RANO^8^ calculation compared to manual calculation Automated volume assessment compared to volume assessment based on manual segmentation	a. Brain extraction: DSC (95% CI) = 0.94 (0.92–0.95) (Dataset 3 test) b. FLAIR segmentation: DSC = 0.80 (0.75–0.80) & volume ICC = 0.92 (*P* < .001) (Dataset 1 test); DSC = 0.82 (0.79–0.84) & volume ICC = 0.92 (*P* < .001) (Dataset 1 external test); DSC = 0.70 (0.67–0.73) & volume ICC = 0.92 (*P* < .001) (Dataset 2 test) c. ET segmentation: DSC = 0.70 (0.66–0.73) & volume ICC = 0.97 (*P* < .001) (Dataset 2 test)	a. FLAIR volume longitudinal change ICC = 0.92 (*P* < .001) (Dataset 2 test) b. ET volume longitudinal change ICC = 0.97 (*P* < .001) (Dataset 2 test) c. RANO bi-dimensional product (ET) ICC range 0.50–0.77 (*P* < .001) (individual rater differences) (Dataset 2 test) d. RANO bi-dimensional product (ET) longitudinal change ICC = 0.85 (*P* < .001) (combined raters) (Dataset 2 test)
Nalepa, et al.^23^	Dataset 1 BraTS 2020 pre-operative dataset: 1. Grade 4 glioma (Glioblastoma) (WHO, 2021) 2. Retrospective 3. Multisite 4. T1, T1 C, T2, FLAIR 5. 660 patients with 660 MRIs (369 train; 125 validate; 166 test) Dataset 2 Phase 3 cohort: 1. Grade 4 glioma (Glioblastoma) (WHO, 2021) 2. Retrospective 3. Multisite 4. T1, T1 C, T2, FLAIR 5. 100 patients with 100 pre-operative MRIs (100 train) 504 patients with 504 post-operative MRIs (464 train; 40 test)	1. Preprocessing a. Co-registration b. Skull-stripping c. Resampling 2. Segmentation 3. RANO^6^-based bi-dimensional product calculation	Confidence-aware nnU-Net compared to manual segmentation Automated RANO^6^ calculation compared to manual calculation Automated volume assessment compared to volume assessment based on manual segmentation	a. Segmentation performance for ET (Dataset 1 validate and test) mean DSC = 0.744 (95%CI = 0.690–0.799); mean H95 = 39.624 mm b. Segmentation performance (Dataset 2 test) mean DSC (95% CI) = 0.692 (0.628–0.757), 0.677 (0.631–0.724) and 0.691 (0.604–0.778) for ET, ED, and surgical cavity; mean H95 (25p–75p) = 9.221 mm (6.437–12.000 mm), 9.455 mm (7.176–11.730 mm) and 7.956 mm (5.938–9.975 mm) for ET, ED, and surgical cavity d. Automatic segmentation volumetric measurements agreement with GT (Dataset 2) ICC (ET): 0.959 mm³ (*P* < .001) ICC (cavity): 0.960 mm³ (*P* < .001) ICC (ED): 0.703 mm³ (*P* < .703)	a. Inter-rater agreement for RANO bidimensional measurements (Dataset 2 test) manual RANO compared to automated RANO (Diameters) (ET) ICC = 0.299–0.866 (*P* < .001) manual RANO compared to automated RANO (Product) (ET) ICC = 0.292–0.858 (*P* < .001) maximum manual RANO compared to automated RANO (Diameters) ICC: 0.915 (*P* < .001) maximum manual RANO compared to automated RANO (Product) ICC: 0.919 (*P* < .001)
Vollmuth, et al.^24^	Dataset 1 Heidelberg Cohort: 1. Grade II-IV glioma (pre 2021 WHO classification) 2. Retrospective 3. Single-site 3. T1, T1 C, T2, FLAIR, DWI, ADC 4. 30 patients with 450 pairs assessment results	1. Preprocessing a. Skull-stripping b. Co-registration c. T1 subtraction 2. Segmentation 3. Calculation of TTP	Automated nnU-Net based segmentation and modified RANO^8^ assessment compared to manual segmentation and assessment AI-based TTP assessment compared to Manual TTP assessment	N/A	a. TTP Assessment comparison between investigators using AI assistance (95% CI): CCC = 0.91 (0.82–0.95) (*P* = .005) (Dataset 1) b. LGG TTP (95% CI): CCC = 0.90 (0.76–0.95) (*P* = .008) (Dataset 1) c. Glioblastomas TTP (95% CI): CCC = 0.83 (0.75–0.92) (*P* = .016) (Dataset 1) d. SD TTP Measurements (95% CI): 4.8 months (3.7–6.2 months) (*P* = .004) (Dataset 1) e. SD LGG TTP (95% CI) = −1.7 months (−4.2 to −1.1 months) (Dataset 1) f. SD Glioblastoma TTP (95% CI) = −0.1 months (−0.5 to 0.0 months) (*P* < .001) (Dataset 1)
Rudie, et al.^25^	Dataset 1 BraTS 2020 pre-operative dataset: 1. Grade 4 glioma (Glioblastoma) (WHO, 2021) 2. Retrospective 3. Multisite 4. T1, T1 C, T2, FLAIR 5. 369 patients with 369 MRIs (All for training an initial segmentation network Dataset 2 Retrospective posttreatment cohort: 1. Grade 2-4 glioma (WHO, 2021) 2. Retrospective 3. Single-site 4. T1, T1 C, T2, FLAIR 5. 298 patients (198 train; 100 test) with 596 MRIs	1. Preprocessing a. DICOM to NifTI conversion b. Registration c. 1 × 1 × 1 interpolation d. Skull-stripping e. Bias correction 2. Additional preprocessing between two timepoints for longitudinal change networks a. Registration b. Subtraction 3. Segmentation 4. Longitudinal volumetric change classification	nnU-Net segmentation network compared to 3D U-Net trained only on the dataset 1 nnU-Net longitudinal change network compared to attending neuroradiologists manual longitudinal volumetric change classification	a. Segmentation network (Dataset 2 test) [Mean ± SD; Median with 25%–75% IQRs] WT: DSC = [0.86 ± 0.10; 0.89 (0.84–0.93)]; Volume Similarity = [0.94 ± 0.10; 0.96 (0.92–0.98)]; HD95 (mm) = [6.9 ± 10.0; 3.3 (1.7–7.1.0)] ED: DSC = [0.85 ± 0.11; 0.8 (0.83–0.92)]; Volume Similarity = [0.94 ± 0.09; 0.96 (0.92–0.99)]; HD95 (mm) = [6.6 ± 10.1; 3.0 (1.4–6.7)] TC: DSC = [0.71 ± 0.27; 0.82 (0.55–0.92)]; Volume Similarity = [0.82 ± 0.25; 0.95 (0.74–0.98)]; HD95 (mm) = [8.6 ± 14.6; 10.4 (1.4–8.3)] ET: DSC = [0.71 ± 0.26; 0.82 (0.55–0.92)]; Volume Similarity = [0.83 ± 0.25; 0.96 (0.80–0.99)]; HD95 (mm) = [8.2 ± 14.7; 10.4 (1.0–7.9)] NCR: DSC = [0.65 ± 0.29; 0.72 (0.49–0.88)]; Volume Similarity = [0.80 ± 0.26; 0.90 (0.74–0.97)]; HD95 (mm) = [5.9 ± 8.1; 10.4 (1.4–6.0)]	a. Longitudinal change network (Dataset 2 test) [Mean ± SD; Median with 25%–75% IQRs] ED change: DSC = [0.73 ± 0.25; 0.83 (0.64–0.88)]; Volume Similarity = [0.84 ± 0.27; 0.94 (0.85–0.98)]; HD95 (mm) = [10.3 ± 11.6; 5.7 (2.0–15.1)] ET change: DSC = [0.60 ± 0.26; 0.67 (0.45–0.81)]; Volume Similarity = [0.73 ± 0.27; 0.86 (0.68–0.92)]; HD95 (mm) = [14.2 ± 16.9; 5.4 (2.5–19.4)] b. Longitudinal classification performance for 3 classes (Dataset 2 test) ED Longitudinal change network: Sensitivity = 0.91; Specificity = 0.91; PPV = 0.89; NPV = 0.93; F1 = 0.85; Accuracy = 0.91; *P* = .84 ET Longitudinal change network: Sensitivity = 0.88; Specificity = 0.92; PPV = 0.88; NPV = 0.92; F1 = 0.88; Accuracy = 0.90; *P* = .61 c. Longitudinal classification performance for 2 classes (Dataset 2 test) ED Longitudinal change network: Sensitivity = 0.94; Specificity = 0.93; PPV = 0.86; NPV = 0.97; F1 = 0.90; Accuracy = 0.93; *P* = .81 ET Longitudinal change network: Sensitivity = 0.87; Specificity = 0.94; PPV = 0.87; NPV = 0.94; F1 = 0.87; Accuracy = 0.92; *P* = .48
Strack, et al.^26^	Dataset 1 Local dataset: 1. Grade IV glioma (glioblastoma) (pre 2021 WHO classification) 2. Retrospective 3. Single-site 4. T1 C 5. 15 patients Dataset 2 TCIA dataset: 1. Grade IV glioma (glioblastoma) (pre 2021 WHO classification) 2. Retrospective 3. Multisite 4. T1 C 5. 20 patients with 40 MRIs	1. Preprocessing a. Resampling b. Histogram matching c. Normalization d. Brain centering e. Skull-stripping 2. Augmentation a. Shifting b. Rotation c. Gaussian noise 3. Segmentation by BraTS model 4. Wasserstein GANs learning changes between time 1 and time 2 images 5. Modified RANO-based classification according to ET volume change	Automated volume change assessment and classification based on Wasserstein GANs compared to manual volume assessment and modified RANO^8^ classification	N/A	ROC analysis of tumor change micro-average AUC = 0.87 (Dataset 1 & 2); total tumor growth AUC = 0.87 (Dataset 1); total AUC = 0.86 (Dataset 2); tumor growth AUC = 0.72 (Dataset 1); tumor reduction AUC = 0.75 (Dataset 1); tumor growth AUC = 0.94 (Dataset 2); tumor reduction AUC = 0.94 (Dataset 2) b. RANO classification overall sensitivity = 0.66 (Dataset 1 & 2); overall specificity = 0.83 (Dataset 1 & 2); total accuracy = 0.66 (Dataset 1 & 2); sensitivity = 0.65 (Dataset 1); specificity = 0.82 (Dataset 1); sensitivity = 0.64 (Dataset 2); specificity = 0.82 (Dataset 2)
Jalalifar, et al.^27^	Dataset 1: 1. Brain metastasis 2. Retrospective 3. Single-site 4. T1 C, FLAIR 5. 116 patients with 152 tumors (train: 96 patients with 130 tumors; independent test: 20 patients with 22 tumors)	1. Preprocessing a. Resampling b. Voxel intensity normalization c. Co-registration 2. Segmentation 3. Calculating the changes of longest diameters and volume 4. Treatment response classification as shrinkage/steady/enlargement 5. Automatic detection of LC/LF and ARE outcome	A proposed combination model of 2D U-Nets, 3D U-Net and MSGA for segmentation compared to manual segmentation by expert oncologists Automated longest diameters and volume assessment compared to manual longest diameters assessment based on RANO-BM^7^ and volumetric assessment criteria Automatic detection of LC/LF and ARE outcome compared to manual assessments by expert oncologists.	a. Tumor segmentation of baseline and follow-up scans (Dataset 1 independent test) DSC = [0.84 ± 0.07, 0.92 ± 0.04] HD95 (mm) = [2.1 ± 0.6, 3 ± 0.6] VEE (cc) = [0.44 ± 0.4, 0.62 ± 0.6] VEE = [0.10 ± 0.05, 0.20 ± 0.09]	a. Tumor size status detecting (Dataset 1 independent test) Accuracy = 0.86; Precision (Increase) = 0.90; Precision (Stable) = 0.75; Precision (Decrease) = 1.00; Recall (Increase) = 0.90; Recall (Stable) = 0.91; Recall (Decrease) = 0.76 b. Tumor response assessments by longest diameter of tumor (Dataset 1 independent test) Accuracy = 0.84; (Enlargement, PD) Precision = 0.78; (Steady, SD) Precision = 0.92; (Shrinkage, PR) Precision = 0.82; (Enlargement, PD) Recall = 0.90; (Steady, SD) Recall = 0.82; (Shrinkage, PR) Recall = 0.82 c. Tumor response assessments by tumor volume (Dataset 1 independent test) Accuracy = 0.81; (Enlargement, PD) Precision = 0.76; (Steady, SD) Precision = 0.86; (Shrinkage, PR) Precision = 0.80; (Enlargement, PD) Recall = 0.80; (Steady, SD) Recall = 0.89; (Shrinkage, PR) Recall = 0.71 d. Detecting LC/LF and ARE outcomes by RANO-BM^7^ (Dataset 1 independent test) Accuracy (LC/LF) = 0.91; Sensitivity (LC/LF) = 0.89; Specificity (LC/LF) = 0.92; Accuracy (ARE) = 0.91; Sensitivity (ARE) = 1.00; Specificity (ARE) = 0.89
Kickingereder, et al.^28^	Dataset 1 Heidelberg training dataset: 1. Grade II-IV glioma (pre 2021 WHO classification) 2. Retrospective 3. Single-site 4. T1, T2, T1 C, FLAIR 5. 455 patients with 455 MRIs (five-fold) Dataset 2 Heidelberg independent test dataset: 1. Grade II-IV glioma (pre 2021 WHO classification) 2. Retrospective 3. Single-site 4. T1, T2, T1 C, FLAIR 5. 40 patients with 239 MRIs Dataset 3 Heidelberg simulation dataset: 1. Grade II-IV glioma (pre 2021 WHO classification) 2. Retrospective 3. Single-site 4. T1, T2, T1 C, FLAIR 5. 466 patients with 595 MRIs Dataset 4 EORTC-26101 external testing dataset: 1. Grade IV glioma (glioblastoma) (pre 2021 WHO classification) 2. Prospective 3. Multisite 4. T1, T2, T1 C, FLAIR 5. 532 patients with 2034 MRIs	1. Preprocessing a. DICOM to NifTI conversion b. Reorientation c. Skull-stripping d. Registration e. T1 subtraction 2. Segmentation 3. Tumor response classification and TTP calculation	U-Net-based model for segmentation compared to manual segmentation Automated volume assessment compared to manual volume assessment based on RANO in 2010^6^ Automated TTP calculation compared to manual TTP assessment	a. CE segmentation agreement DSC (95% CI) = 0.89 (0.86–0.90) (Dataset 2); DSC (95% CI) = 0.91 (0.90-0.92) (Dataset 4) b. CE volume agreement DSC (95% CI) = 0·99 (0·99–1.00) (Dataset 2); DSC (95% CI) = 0.99 (0.99–0.99) (Dataset 4) c. NE segmentation agreement DSC (95% CI) = 0.93 (0.92–0.94) (Dataset 2); DSC (95% CI) = 0.93 (0.93–0.94) (Dataset 4) d. NE volume agreement DSC (95% CI) = 0.99 (0.99–0.99) (Dataset 2); DSC (95% CI) = 0.98 (0.98–0.99) (Dataset 4) e. Concordance Correlation Coefficients ≥ 0.98 (Dataset 2 & 4)	a. Agreement in quantitative volumetrically defined TTP is 0.90, *P* = .94 (Dataset 2) and 0.87, *P* = .77 (Dataset 4) Note: no RANO assessment evaluation
Chen, et al.^29^	Dataset 1 Longitudinal dataset: 1. Brain metastases 2. Retrospective 3. Single-site 4. T1 5. 85 patients with 170 MRIs	1. BMs detection 2. Calculating changes in volume and number of BM lesions	FPN-based CAD of United Imaging Intelligence (uAI) Discover-BMs software compared to manual detection and volume change measurement Automated volume and number of BMs lesion change measurement compared to manual measurement based on RANO-BM^7^	a. Metastasis lesions detection (Dataset 1) Sensitivity = 0.99; FNs (per scan) = 0.06; FPs (per scan) = 0.53; X^2 = 31.15, *P* < .05 b. Follow-up metastasis lesions detection (Dataset 1) Sensitivity = 0.98; FNs (per scan) = 0.08; FPs (per scan) = 0.39; X^2 = 21.09, *P* < .05	a. Agreement of treatment response between automated and manual assessment (Dataset 1): kappa = 0.941, *P* < .05
Meier, et al.^30^	Dataset 1 Longitudinal dataset: 1. Grade IV glioma (glioblastoma) (pre 2021 WHO classification) 2. Prospective 3. Single-site 4. T1, T1 C, T2, FLAIR 5. 14 patients with 64 MRIs	1. Preprocessing by BraTumIA a. Skull-stripping b. Intermodality registration c. Bias field correction 2. Voxel-wise segmentation for NCE-T2 and ET-T1 C by BraTumIA 3. Volume change measurement	Machine learning based BraTumIA software segmentation compared to manual segmentation Automated volume changes assessment by BraTumIA compared to manual volume changes assessment	a. Volume correlations betweent BraTumIA and raters (Dataset 1) *r*-values 0.95 to 0.96, *P* < .001 b. Relative over- or underestimation of the volumes (Dataset 1) BraTumIA compared to human rater 1 0.52 to 9.9	a. Volume change correlations between BraTumIA and raters (Dataset 1) *r*-values 0.83 to 0.96, *P* < .001
Preetha, et al.^31^	Dataset 1 Heidelberg cohort: 1. Grade IV glioma (glioblastoma) (pre 2021 WHO classification) 2. Retrospective 3. Single-site 4. T1, T1 C, T2, FLAIR, ADC 5. 775 patients with 775 MRIs Dataset 2 CORE longitudinal cohort: 1. Grade IV glioma (glioblastoma) (pre 2021 WHO classification) 2. Prospective 3. Multisite 4. T1, T1 C, T2, FLAIR, ADC 5. 260 patients with 1083 MRIs Dataset 3 CENTRIC longitudinal cohort: 1. Grade IV glioma (glioblastoma) (pre 2021 WHO classification) 2. Prospective 3. Multisite 4. T1, T1 C, T2, FLAIR, ADC 5. 505 patients with 3147 MRIs Dataset 4 EORTC-26101 longitudinal cohort: 1. Grade IV glioma (glioblastoma) (pre 2021 WHO classification) 2. Prospective 3. Multisite 4. T1, T1 C, T2, FLAIR, ADC 5. 521 patients with 1924 MRIs	1. Preprocessing a. DICOM to NIfTI conversion b. Reorientation c. Skull-stripping d. Registration e. Resampling f. Normalization g. T1 Subtraction 2. Synthetic T1 C imaging generation 3. ET segmentation map generation 4. ET volume change calculation	The combination model of U-Net and CGAN for generation, segmentation, calculation based on RANO in 2010^6^ compared to manual corresponding assessment	a. Comparison on automated and manual T1 subtraction generation (Dataset 4) CGAN-SSIM (95% CI) = 0.818 (0.817–0.820), *P* < .0001 U-Net-SSIM (95% CI) = 0.809 (0.807–0.810), *P* < .0001 b. Agreement in CE segmentations and volumes between automatic and manual assessment (Dataset 4) CCC (95% CI) = 0.782 (0.751–0.807), *P* < .0001 Spatial agreement Sørensen–DSC: *r* (95% CI) = 0.438 (0.401–0.475), *P* < .0001	a. Agreement in TTP: comparison based on automated and manual T1 subtraction generation assessments (Dataset 4) automated 4.2 months (95%CI 4.1–5.2) manual 4.3 months (95%CI 4.1–5.5) *P* = .33
Cho, et al.^32^	Dataset 1 SNUBH training data: 1. Brain metastases 2. Retrospective 3. Single-site 4. T1, T1 C, T2, FLAIR 5. 174 patients with 127 MRIs Dataset 2 SNUBH temporal test set #1: 1. Brain metastases 2. Retrospective 3. Single-site 4. T1, T1 C, T2, FLAIR 5. 40 patients with 20 MRIs Dataset 3 SNUBH temporal test set #2: 1. Brain metastases 2. Retrospective 3. Single-site 4. T1, T1 C, T2, FLAIR 5. 12 MRIs Dataset 4 SNUH external geographic test: 1. Brain metastases 2. Retrospective 3. Multisite 4. T1, T1 C, T2, FLAIR 5. 24 patients with BM and 11 patients without BM	1. Preprocessing a. Normalization b. Isotropic reconstruction 2. Brain segmentation 3. Brain parenchyma extraction 4. BM detection using 3D U-Net 5. BM segmentation using 2D U-Net (DenseNet 201) 6. 3D rigid registration 7. Volumetric changes calculation	DL-CAD compared to MD for segmentation, detection Automated compared to manual RANO-BM^7^ (the changes of the sum in longest diameters) and volumetric response criteria	a. BM detection Sensitivity (95% CI) = 0.58 (0.53–0.63); DSC = 0.67 ± 0.23; FP/scan = 2.50 (Dataset 2) Sensitivity (95% CI) = 0.80 (0.61–0.92); DSC = 0.76 ± 0.26; FP/scan = 2.20 (Dataset 3) Sensitivity (95% CI) = 0.76 (0.66–0.84); DSC = 0.66 ± 0.22; FP/scan = 7.60 (Dataset 4) b. BM measuring >= 5mm Sensitivity (95% CI) = 0.75 (0.70–0.80); DSC = 0.69 ± 0.22; FP/scan = 0.80 (Dataset 2) Sensitivity (95% CI) = 0.95 (0.74–1.00); DSC = 0.82 ± 0.20; FP/scan = 0.50 (Dataset 3) Sensitivity (95% CI) = 0.88 (0.77–0.95); DSC = 0.68 ± 0.20; FP/scan = 1.90 (Dataset 4)	a. Agreement of the response assessment in RANO-BM and volumetry (Dataset 2 & 3 & 4) RANO-BM measurement: k (95% CI) = 0.52 (0.26–0.79); volumetric measurement: k (95% CI) = 0.68 (0.41–0.94)
Hsu, et al.^33^	Dataset 1: 1. Brain metastases 2. Retrospective 3. Single-site 4. T1 C 5. 20 patients Dataset 2: 1. Brain metastases 2. Retrospective 3. Single-site 4. T1 C 5. train 409 patients with 1345 BMs; test 102 patients with 367 BMs Dataset 3: 1. Brain metastases 2. Retrospective 3. Single-site 4. T1 C 5. 32 patients with 123 BMs	1. BM registration 2. BM segmentation 3. BM tracking 4. Response assessment measurements by 3LD and ESD	Metastasis Tracking with Repeated Observations 3D CNN-based software compared to manual assessments for registration, segmentation, the changes of volume and diameters measurements	a. The average shift across all points of registration 1.5 ± 0.2 mm (95% CI) (Dataset 1) b. Segmentation performance (Dataset 2 test) Sensitivity (95% CI) = 95% ± 3%; False positive rate (95% CI) = 2.4 ± 0.5 per patient; DSC (95% CI) = 0.76 ± 0.03	a. Detection rate of new or unirradiated BMs 72% (Dataset 3) b. Correlation of size responses R² = 0.80 (Dataset 3) c. Pearson correlation coefficient for size changes of non-disappeared lesions was 0.88 for 3LD (3-dimensional longest diameter) and 0.86 for ESD (equivalent spherical diameter), with *P*< .001 (Dataset 3)
Kleesiek, et al.^34^	Dataset 1 Longitudinal data: 1. Grade IV glioma (glioblastoma) (pre 2021 WHO classification) 2. Retrospective 3. Single-site 4. T1, T1 C, T2, FLAIR 5. 15 patients with 71 MRIs Dataset 2 Brats 2013: 1. Grade II–IV gliomas (pre 2021 WHO classification) 2. Retrospective 3. Multisite 4. T1, T1 C, T2, FLAIR 5. 30 MRIs	1. Preprocessing for dataset 1 a. N3 bias field correction b. Resampling c. Longitudinal registration d. Skull-stripping e. Brain mask generation f. Intra-individual registration g. Brain mask application 2. Preprocessing for dataset 2 a. N3 bias field correction b. Normalization 3. Preprocessing for both datasets a. T1 Subtraction b. Feature Extraction 4. Volumetric segmentation 5. Volumetric assessment classification	Random forest-based segmentation and volumetric measurements compared to manual volumetric and RANO in 2010^6^ measurements	a. GTV segmentation DSC = 0.636 (Dataset 1) DSC = 0.963 (Dataset 2)	a. The change of GTV with the virtual raters correlation *r* = .995, *P* < .0001 (Dataset 1)
Ozkara, et al.^35^	Dataset 1 Longitudinal dataset: 1. Brain metastases 2. Retrospective 3. Single-site 4. T1 C 5. 180 patients	1. Tumor segmentation by DL-based algorithm 2. Calculation of volume and longest diameters change measurement by thresholding functions	Automation ML-based software Jazz for segmentation and volumetric assessment compared to manual measurement based on RANO-BM^7^ (the change of longest diameter and volume)	N/A	a. The agreement of volume changes measurement (Dataset 1) ICC = 0.98 (95% CI, 0.97–0.98)
Suter, et al. 2023^36^	Dataset 1 LUMERE post-operative Dataset: 1. Grade IV glioma (glioblastoma) (pre 2021 WHO classification) 2. Retrospective 3. Single-site 4. T1, T1 C, T2, FLAIR 5. 80 patients with 502 MRIs Dataset 2 Scans identified from Dataset 1 containing target lesions: 129 MRIs	1. Preprocessing a. Resampling b. Skull-stripping 2. Segmentation 3. Automated 2D measurement (the product of longest perpendicular diameters in the axial space) 4. Automated volumetric (quantifying the contrast enhancement volume by counting the voxels of the segmentation label) 5. Automated 2.5D measurement (the product of the longest diameters in the tumor 3D space) 6. Classification of treatment response 7. Calculation of TTP	DL-based BraTumIA software and HD-GLIO compared to manual assessments for segmentation, modified RANO^8^ measurements, classification and TTP calculation	N/A	a. Agreement of 2D measurements with manual measurements (Dataset 2) HD-GLIO: 0.81, BraTumIA: 0.80
Zhang, et al.^37^	Dataset 1 Longitudinal dataset: 1.Glioblastoma (pre 2021 WHO classification) 2. Retrospective 3. Single-site 4. T1, T1 C, T2, FLAIR 5. 634 patients with 3403 MRIs	1. Preprocessing a. Field-of-View Standardization b. Skull-stripping 2. Segmentation 3. Volumetric measurements 4. BT-RADS classification	nnU-Net based segmentation to manual segmentation and volumetric assessments compared to volumetric assessments based on BT-RADS (volume)	a. Mean ± SD = 0.8861 ± 0.2476 for enhancing tumor and 0.9833 ± 0.0372 for surrounding non-enhancing FLAIR signal abnormality (Dataset 1 internal validation test)	a. The agreement across BT-RADS (F1: 0.587–0.755) (Dataset 1 internal test) b. Kaplan–Meier Survival Analysis: Worse survival for human-assessed progression vs. AI (Log-rank *P* = .007) (Dataset 1 internal test) c. Cox Proportional Hazard Model Analysis: AI assessments less accurate for survival prediction (*P* = .012) (Dataset 1 internal test)
Prezelski, et al.^38^	Dataset 1 Longitudinal dataset: 1. Brain metastases 2. Retrospective 3. Single-site 4. T1 5. 71 patients with 176 BMs, 629 MRIs	1. BM detection 2. Segmentation 3. Rigid registration 3. BM volume and longest 3D diameter changes calculation 4. BM classification	Metastasis Tracking with Repeated Observations 3D CNN-based software compared to manual assessments for detection, segmentation, the caculation of volume and longest 3D diameter changes and classification based on RANO-BM^7^	a. BM detection (Dataset 1) Sensitivity: Nearly 100% for larger lesions, drops below 90% for lesions smaller than 5 mm	a. BM volume and 3LD changes Calculation (Dataset 1) Correlation Coefficient (R^2^): 0.76 (*P* = .0001) Comparison between Manual and METRO Measurements: METRO’s longest 3D diameter is generally longer than the manual axial diameter b. BM classification (Dataset 1) Sensitivity: 0.72 Specificity: 0.95 Precision: 0.81 (Increasing), 0.32 (Stable), 0.36 (Decreasing), 0.66 (Unappreciable) Recall: 0.72 (Increasing), 0.55 (Stable), 0.22 (Decreasing), 0.72 (Unappreciable) Specificity: 0.95 (Increasing), 0.82 (Stable), 0.85 (Decreasing), 0.77 (Unappreciable) F1-score: 0.76 (Increasing), 0.40 (Stable), 0.27 (Decreasing), 0.69 (Unappreciable)
Son, et al.^39^	Dataset 1 Segmentation dataset: 1. Brain metastases 2. Retrospective 3. Single-site 4. T1, T1 C, T2, FLAIR, BB T1 5. 128 patients with 1339 BMs Dataset 2 Treatment response dataset: 1. Brain metastases 2. Retrospective 3. Single-site 4. T1, T1 C, T2, FLAIR, BB T1 5. 58 patients with 629 BMs	1. Skull-stripping 2. BM detection 3. BM segmentation 4. Volumetric changes caculation 4. RANO-BM^7^ classification	RLK-UNet based architecture compared to manual assessments for detection, segmentation, volume changes changes and classification based on RANO-BM^7^	a. Detection performance: (Dataset 1) Sensitivity: 86.9% Precision: 79.6% False Positives per Scan: 1.76 b. Segmentation performance: (Dataset 2) All BMs (DSC): 0.663 Large BMs (DSC): 0.851 Small BMs (DSC): 0.535 Pearson Correlation Coefficient: 0.96 Bland-Altman analysis: Mean difference of 0.01 cm³	a. Agreement on treatment response assessment: (Dataset 2) ICC: 0.84 (95% CI: 0.75–0.91) b. Agreement in response assessment: 87.9% (51/58 patients) Overestimation of treatment response: 6.8% (4/58 patients) Underestimation of treatment response: 5.1% (3/58 patients)
Kotowski, et al.^40^	Dataset 1 BraTS2021 training dataset: 1. Glioblastoma (pre 2021 WHO classification) 2. Retrospective 3. Multisite 4. T1, T1 C, T2, FLAIR 5. 1251 patients with 1251 MRIs Dataset 2 Brain tumor progression dataset: 1. Glioblastoma (pre 2021 WHO classification) 2. Retrospective 3. Multisite 4. T1, T1 C, T2, FLAIR 5. 20 patients with 40 MRIs	1. Preprocessing by CaPTK a. Reorientation b. Resampling c. Denoising d. Bias correction e. Co-registration 2. Skull-stripping 3. Segmentation 4. Bidimensional and volumetric measurements	HD-BET 3D U-Net based brain extraction, nnU-Net segmentation, AutoRANO and volumetric measurement compared to manual measurement based on RANO 2010^6^	a. Segmentation DSC: (Dataset 2) DeepMedic: mean 0.72, median 0.77 (95% CI: 0.66–0.79) HD-BET: mean 0.73, median 0.79 (95% CI: 0.67–0.80)	a. Bidimensional measurements spearman’s correlation coefficient: (Dataset 2) DeepMedic: 0.58 HD-BET: 0.68 b. Measurable ET Volume Correlation Coefficient: (Dataset 2) DeepMedic: 0.90 HD-BET: 0.93 c. Full ET Volume Correlation Coefficient: (Dataset 2) DeepMedic: 0.89 HD-BET: 0.93
Hammer, et al.^41^	Dataset 1 D-STUDIES dataset: 1. Brain metastases 2. Retrospective 3. Single-site 4. T1 C 5. 226 patients Dataset 2: D-SCANS dataset (created from Dataset 1 for time-sequenced analysis and pairing creation) 1. Brain metastases 2. Retrospective 3. Single-site 4. T1 C 5. 226 patients with 500 MRIs Dataset 3: D-SCAN-PAIRS dataset (created by pairing pre-SRS with post-SRS scans) 1. Brain metastases 2. Retrospective 3. Single-site 4. T1 C 5. 271 pairs of time-ordered MRI scans (train/validate 205 pairs from 169 patients; test: 66 pairs from 57 patients) Dataset 4: D-LESIONS-GT dataset (Dataset 2 annotations) 1. Brain metastases 2. Retrospective 3. Single-site 4. 1,889 lesions annotated (From 439 scans:1,571 lesions manually annotated by experts; From 61 scans: 318 lesions refined from SimU-Net predictions) Dataset 5: D-LESION-PAIRS-GT dataset (Dataset 3 annotations) 1. Brain metastases 2. Retrospective 3. Single-site 4. 2,055 lesions annotated: (Includes lesions from pre-SRS scan repetitions)	1. Brain segmentation 2. Registration 3. Simultaneous lesion detection and segmentation 4. Detection and classification of lesion changes 5. Quantification	SimU-Net based detection, segmentation, classification compared to manual measurements	a. Lesion Detection (Dataset 3 test): (Lesions > 10 mm) Precision: 1.00 ± 0.00 Recall: 1.00 ± 0.00 (Lesions > 5 mm) Recall (all scenarios): 0.95–0.96 ± 0.13–0.14 Simultaneous without prior & Standalone pairs (Precision): 0.92–0.93 ± 0.18–0.19 Other scenarios (Precision): 0.86–0.89 ± 0.24–0.28 (Lesions of All Sizes) Standalone scenarios (Recall): 0.82–0.83 ± 0.28–0.29 Other scenarios (Recall): ~0.80 ± 0.28–0.31 Simultaneous with prior (Precision): 0.83 ± 0.24 Other scenarios (Precision): 0.75–0.78 ± 0.26–0.28 b. Lesion Segmentation (Dataset 3 test) DSC: 0.80–0.90 ± 0.10–0.21 ASSD: 0.27–0.62 ± 0.35–1.27 mm Simultaneous without prior: DSC 0.83–0.90 ± 0.10–0.22	a. Lesion Matching (Dataset 2) Precision and Recall: 1.00 ± 0.00 b. Lesion Change Classification: (Dataset 2) Precision and Recall: 1.00 ± 0.00

WHO, 2021 (World Health Organization Classification of Tumors of the Central Nervous System in 2021).^20^ T1, T1-weighted; T2, T2-weighted; T1 C, post contrast T1-weighted; FLAIR, fluid-attenuated inversion recovery; DWI, diffusion-weighted imaging; ADC, apparent diffusion coefficient; RANO, response assessment in neuro-oncology; ICC, intraclass correlation coefficient; ET, enhancing tumor; ED, peritumoral edematous, infiltrated, or treatment-changed tissue; NCR, necrotic core; TC, tumor core (AT + NCR); WT, whole tumor (ED + AT + NCR); HD95, Hausdorff 95th percentile distance; TTP, time to progression; CCC, concordance correlation coefficient; LGG, low-grade glioma; SD, standard deviation; AUC, area-under-the-curve; 25p–75p, 25% percentile–75% percentile; GT, ground truth; AUC, area under the curve; LF, local failure; LC, local control; SRS, stereotactic radiotherapy; ARE, adverse radiation effect; PD, progressive disease; PR, partial response; SD, stable disease; RANO, response assessment in neuro-oncology; RANO-BM, response assessment in neuro-oncology-brain metastases; *P*, *P*-value; BMs, brain metastases; CAD, computer-aided detection; MD, manual detection; SSIM, structural similarity index measure; DL, deep learning; BraTS, brain tumor segmentation challenge; ANN, artificial neural network; 3LD, 3D longest diameter; ESD, equivalent sphere diameter; GTVs, gross tumor volume; CI, confidence interval; DSC, dice similarity coefficient; CaPTK, Cancer Imaging Phenomics Toolkit; ASSD, average symmetric surface distance.

This table synthesizes 20 studies highlighting the datasets used, research designs, and machine learning models employed. It summarizes the automated processes implemented in the pipeline, such as brain extraction, tumor segmentation, and volume measurement. Performance metrics are presented based on published information or calculated from available data—the metrics vary according to task.

### Study Characteristics

As shown in Table 1, in terms of dataset utilization, 60.0% (12/20) of studies included grade 2–4 glioma, with 66.7% (8/12) of studies focusing solely on glioblastoma (studies used grade IV glioma WHO 2016 definition). The remainder, 40.0% (8/20) of studies, focused on brain metastases.

Studies differed as to whether they were either validation-only studies or combined development and validation studies. Of 50.0% (10/20) studies that trained ML models and conducted tests with hold-out data, all (100% 10/10) studies utilized internal test data and 40.0% (4/10) studies additionally employed external test data. In the remaining studies, 5.0% (1/20) only used external test data, and 45.0% (9/20) did not involve model training (ie, the latter were validation-only studies where the ML model had been developed in a previous study).

There were 55.0% (11/20) studies using multi-institutional data. Few studies incorporated prospective data (15.0%, 3/20). 60.0% (12/20) of studies used post-operative data only for training or testing, while 40.0% (8/20) of studies trained segmentation models using predominantly pre-operative data or combined with a little post-operative data, and then applied the trained model to post-operative testing data. Such a strategy is expedient because pre-operative datasets are arguably more abundant and accessible than post-operative datasets; pre-operative datasets were typically from the Brain Tumor Segmentation (BraTS) Challenge.^42^ Despite the benefits of expediency, while current results of test data might be acceptable, the domain gap of such an approach leads to risks of poor generalizability.

Most studies (70.0%, 14/20) employed more than one MRI sequence. Structural imaging sequences (T1-weighted (T1), T1-weighted post-contrast (T1 C), T2-weighted (T2), and FLAIR) were predominantly used, with T1 C applied in 90.0% (18/20) of the studies, in keeping with its pivotal role in therapeutic response assessment.^6,43^ Some studies 10.0% (2/20) also used diffusion-weighted imaging (DWI) or apparent diffusion coefficient (ADC) maps.^44^

Regarding implementation methodologies, while our inclusion criteria required employing some form of ML-based automation, the extent of automation varied with 80.0% (16/20) of studies achieving complete automation from start to finish without manual intervention. The remainder (20.0%, 4/20) used semi-automatic frameworks with manual intervention.

Most studies (85.0%, 17/20) focused on advanced ML algorithms by utilizing deep neural networks for learning and inference, such as employing a 3D U-Net-based model^22,23^ for segmentation assessment. The remainder, 15.0% (3/20), did not use deep learning.

In longitudinal assessment, most studies (75.0%, 15/20) employed RANO-based criteria, calculating the diameter and/or volume change, as the assessment method for evaluating therapeutic response. Amongst these, 40.0% (6/15) were based on brain metastases RANO (RANO-BM),^7^ 33.3% (5/15) were based on high-grade glioma RANO (criteria from 2010),^6^ and 26.7% (4/15) on modified high-grade glioma RANO (criteria from 2017 where a key difference is that the baseline MRI is the one performed soon after radiotherapy completion).^8^ In terms of measurements, 66.7% (10/15) studies utilized diameter measurement methods, with 30.0% (3/10) employing “RANO 2010,”^6^ 30.0% (3/10) employing “modified RANO 2017,”^8^ and 40.0% (4/10) employing RANO-BM^7^ criteria. Following RANO 2010 or modified RANO 2017 criteria,^6,8^ a few of these studies additionally used volume measurement methods (20.0%, 3/15) while the rest (26.7%, 4/15) only employed volume measurement assessments. In the remaining 25.0% (5/20) of studies where RANO-based criteria were not employed, evaluation metrics were solely based on volume change in 60.0% (3/5), while 40.0% (2/5) of studies incorporated both volume and diameter changes for assessment. We also note that 40.0% (8/20) of studies were based on newly diagnosed patients and 30.0% (6/20) on recurrent patients, with the remaining 30.0% (6/20) unknown.

### QUADAS-2 Assessments of the Included Studies

**Figure 2.**
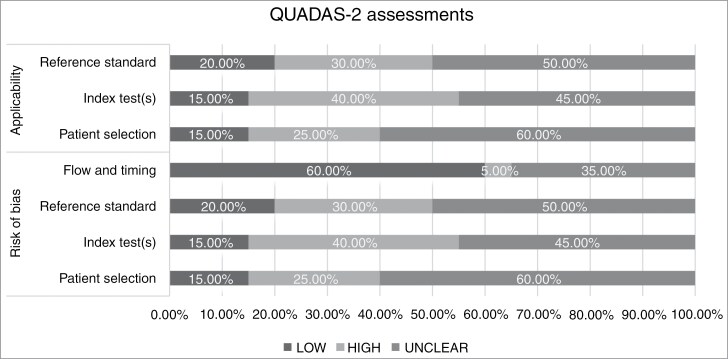
Summary of the QUADAS-2 assessments of the included studies. Graphical representation of included studies (in percentages) in each key domain in terms of the risk of bias and the concerns regarding applicability. each bar signifies the assessed risk levels, with left side of bar chart (blue) indicating “low” risk/concern, mid bar chart (orange) signifying “high” risk/concern, and right bar chart (gray) denoting “unclear” risk/concern.

The results of the QUADAS-2 stratified analysis^18^ of both risk of bias and applicability across 4 domains, (patient selection, index test(s), reference standard, and flow and timing), are presented in Figure 2, Supplementary Tables S2 and Table S4. In terms of risk of bias and concerns regarding applicability, only 15.0% (3/20), 15.0% (3/20), 20.0% (4/20), and 60.0% (12/20) are considered at “low” risk in the domains of patient selection, index test(s), reference standard, and flow and timing, respectively. The corollary is that there is either an “unclear” or “high” risk of bias and applicability concerns in most studies.

When focusing on particular aspects of bias, a more nuanced picture emerges. In the patient selection domain, we observed that the majority of studies (50.0%, 10/20) had included patients undergoing a clearly stated treatment protocol (eg, Stupp protocol for glioblastoma)^1,45^; 45.0% (9/20) studies explicitly stated that patients were enrolled as either a consecutive or random sample; and half the studies (60.0%, 12/20) applied appropriate exclusions (eg, the studies excluded cases without documented original histology or incomplete or poor-quality imaging data). Similarly, several components of good study design reducing bias risk in the index tests and reference standard domains were also evident in many studies. First, researchers were blinded to the reference standard performance when considering index test performance in most studies (90.0%, 18/20)—and vice versa in (80.0%, 16/20) studies. The blinding ensured the reliability of the outcome measurement and reduced the potential for verification bias. Second, it was clearly stated that trained model parameters had been fixed during the testing process in almost all studies (85.0%, 17/20). Third, in all experiments, at least one senior radiologist was involved in manual annotation.

## Discussion

### Summary of Findings

Automated longitudinal treatment response assessment of brain tumors has been achieved for brain metastases, as well as both high- and low-grade gliomas. The prevailing approach remains the development of ML-based methods emulating RANO criteria,^6–8^ with glioblastoma being the commonest tumor to be assessed. When ML-based methods were employed as the index test and compared to the reference standard of expert manual assessments, the performance accuracy was generally good. However, there was a high or unclear risk of bias within most studies due to incomplete published information and a lack of rigor in experimental design, which constrains the widespread applicability of the automated systems. Similarly, there was no clear evidence indicating that the automated systems could be applied in clinical settings. Despite the quality assessment findings, and despite the fact that the studies are generally of a low level of evidence,^46^ there is value in interrogating these individual studies as they represent the current state of the art and form a baseline for further research.

### Study Explanations and Relevance From a National and International Perspective

This is the first systematic review of automated longitudinal treatment response assessment studies for brain tumors. It has shown that the neuro-oncology imaging research community has leveraged the ability to obtain, process, and store digital images, harnessed the improved performance of registration and segmentation tools—and taken together—have built automated treatment response assessment tools. However, while analytical validation^5^ has been demonstrated to be technically possible for a range of brain tumors, almost all current studies are compromised by bias and are best-considered proof-of-concept studies. One recent multi-reader validation study has largely avoided bias,^24^ but the study design does not constitute comprehensive clinical validation^5^ (Box 3). Therefore, from published evidence, no tool is definitively ready for clinical use and more research is required to ensure this. Being clinic-ready is important because once performance accuracy is satisfactory in providing treatment response assessment during either a clinical trial investigating therapeutics or during routine patient follow-up, there is a high likelihood of benefit for both the patient and the healthcare system. The key potential benefit is that a clinically validated tool will reduce or eradicate interobserver error of treatment response assessment by giving a more reproducible and standardized result and that the tool will reduce the burden of time and costs spent on clinical trials. Beyond trials, it is also plausible that a clinically validated tool will improve consistency in routine patient follow-up in the clinic and therefore allow more rational management decisions.

Box 3: **Comprehensive Clinical Validation**

Comprehensive clinical validation would require not only embedding tools within the clinical or trial workflow but also demonstrating robust real-world utility across diverse patient populations and clinical settings. This also includes validation datasets from multiple institutions to ensure generalizability; testing with varied imaging protocols and scanner vendors; and prospective trials that assess clinical outcomes when using these tools in decision-making.

### Limitations—Studies Assessed

While the studies assessed show numerous strengths they are not without their limitations. First, few studies employed external testing limiting the analytical validation process,^5^ so it is unclear whether the tools are generalizable and therefore applicable for further use at other sites. Second, few studies used prospective data, and none employed tools embedded in the clinical or trial workflow, therefore clinical validation^5^ steps are still required. Third, given that RANO 2.0 was only published in 2023, no studies using ML-based automation have yet conducted experiments based specifically on the updated criteria.^9^ Fourth, there is a clear need for more studies to adopt fully automated measurement techniques (diameters and volume changes calculation) to enhance the accuracy and consistency of RANO assessments; at least a quarter are semi-automated. Fifth, RANO criteria,^6–9^ and the Macdonald criteria^13^ that preceded them, replaced the previous World Health Organization (WHO) recommendations^47^ which considered all brain tumors as solid entities during measurement. The Macdonald criteria allowed assessment of tumors within cavity walls but did not distinguish between the presence of necrosis or surgical resection in a “cyst-like” cavity. The RANO criteria indicated that any cyst-like cavity should not be measured. However, numerous studies—including automated longitudinal treatment response assessment tools purporting to follow RANO criteria—often demonstrate the inclusion of some or all cyst-like cavities during tumor measurement.^22–24,34,36^ It is conceivable that global segmentation competitions where concepts such as “entire tumor core” which have included cyst-like cavities by definition, have disproportionately influenced the current models. In the application of RANO criteria^6–8^ as a reference standard, a bidimensional and volumetric measurement systematic error will reduce the accuracy of both the reference standard and the index test.^6–8^ Reproducibility is likely to be impacted too as reference standard systematic errors will likely vary between sites. Sixth, segmentation competitions such as BraTS^42^ have almost always included pre-operative datasets alone; this poses a challenge for developing generalizable models for the use case of longitudinal treatment response which needs at least some post-operative datasets. Incorporating more post-operative data in these challenges—as has occurred in BraTS 2024^42,48^ is meaningful as it better aligns with the assessment of treatment response in clinical practice and future research. Seventh, few studies explicitly stated that patients were enrolled as either a consecutive or random sample, and in approximately half the studies, there was no evidence to demonstrate that all appropriate exclusions were applied. This lack of clarity suggests a potential bias in patient selection, which could limit the generalizability of the research findings. Eighth, when designing the index test, few studies (15.0%, 3/20) explicitly stated that they considered relevant clinical characteristics important for the final longitudinal treatment response assessment. When considering using RANO criteria as a reference standard in glioma, for example, factors such as a change in performance status, a change in the use of corticosteroids, and the start of second-line treatment are essential for final longitudinal treatment response assessment as RANO is a clinico-radiological assessment.^49^ Ninth, there was a mismatch between the proposed RANO study scheme and the actual baseline used in the study; researchers may not have realised that the modified RANO baseline is the first scan after radiotherapy, and the RANO 2010 baseline is after surgery but before radiotherapy.^6,8^

### Limitations—Review Process

This paper is the first systematic review of automated pipelines that assess brain tumor treatment response using ML. However, the review still has some limitations. First, our review did not include those ML studies that only provide theoretical foundations or preliminary data without experiments.^50^ While these studies do not involve direct clinical experiments, it is plausible that they are potentially valuable for the development of longitudinal treatment response assessment tools. Second, we also excluded those automated pipelines without ML even if they were able to assess automated longitudinal treatment response, as they were beyond the remit of the systematic review. Nonetheless for comparison, some important studies that utilize automatic algorithms, such as those using the region-growing algorithm to achieve automation,^51–53^ are shown in Supplementary Table S3. Third, studies were excluded which focused on the development of prognostic biomarkers for overall survival (OS) based on radiographic feature changes.^54^ However, there is some overlap in longitudinal research methodology which might be relevant to treatment response assessment. Fourth, publication bias may also have affected the range of automated pipelines of treatment response included in this systematic review. Related to this, the exclusion of pre-prints and non-peer-reviewed material may exacerbate publication bias. In particular, given that some in the data science community may not submit their work in peer-reviewed journals, as peer review is relatively slow compared to the speed at which data science develops, it is plausible that publication bias relates to the make-up of the researcher team.^12^ For example, more clinically orientated teams may be more inclined to publish in a peer-reviewed journal compared to more data science-orientated teams who sometimes use pre-prints alone or full-length conference proceedings.^12^ Fifth, our search strategy may not have captured all relevant studies.

### Current Evidence in the Field

This is the first systematic review of automated longitudinal treatment response assessment studies for brain tumors. The response evaluation criteria in solid tumors (RECIST) is a widely used reference standard for evaluating the efficacy of therapies in patients with solid tumors which are included in clinical trials and it is widely used and accepted by regulatory agencies.^55^ Similar to brain tumor response assessment using automated RANO assessments, automation of RECIST assessments is desirable as it can potentially streamline the process and potentially reduce the variability of RECIST-based results.^55^ However, there remain technical challenges which must be overcome to ensure reproducibility, and currently, there are no clinic-ready automated RECIST studies to the best of our knowledge. The current evidence suggests that RANO^6–8^ appears more likely to achieve full automation with fewer remaining challenges compared to RECIST.^55^

### Implications for Clinical Practice and Future Research

These automated tools can enhance the overall efficiency of tumor treatment response assessment and reduce interobserver variability. While the main intention for RANO assessments—including when using automated tools—is to produce measurable, standardized, and meaningful outcomes for clinical trials, approximating RANO assessment into routine real-world follow-up assessment may also help clinicians make more reproducible treatment decisions given the complexities of interpreting MRI data.^11^ If further developed and validated, it is plausible that automation might overcome the time-consuming process preventing RANO-like assessments from being routinely available in the clinic. Considering the directions for future developments, attention should be focused on the main aspects detailed hereafter.

First, new automated tool developments incorporating the requirements of criteria like RANO 2.0 (which incorporate bidimensional diameters and volumes, use different contrasts, and allow assessment of both high-grade gliomas and low-grade gliomas^9^), are likely to support future clinical trials and be more translatable to the clinic. Second, adherence to the incorporation of tumor tissue demarcated by T1 C, as opposed to including voluminous cyst-like regions, is needed in ML models to keep RANO assessments as they were intended during inception over a decade ago. Third, to enhance utility, given that treatment response assessment of brain tumors is a clinico-radiological assessment, tool interfaces would benefit from having the option to integrate clinical information such as changes in performance status or steroid use and/or contain user warnings to not confound assessment by not considering confounders such as early second-like treatment^12^ which is a common concern for RANO assessments.^8,9^ Fourth, consideration should also be given to distinguishing target lesions, measurable lesions, and whether lesions are inside or outside the radiotherapy field—all of which are requisites for the assessment of longitudinal treatment responses in RANO.^9^ Fifth, it should be noted that there is ongoing controversy regarding the optimal approach to measuring brain tumors over time. For glioblastomas, it remains unclear whether measurements should focus solely on the enhancing portion or also include non-enhancing FLAIR signal abnormalities. Similarly, for IDH-mutant gliomas, standardized measurement strategies are not yet well-defined. Additionally, as highlighted by RANO 2.0 criteria,^9^ volumetric measurements have not been shown to be unequivocally superior to orthogonal diameters. Furthermore, clinical trials seeking to modify the Stupp protocol may influence the selection of a “post-treatment baseline,” but the implications of these modifications remain uncertain. In summary, the RANO criteria, as articulated from inception in 2010, are best considered as works in progress and will continue to evolve.^6^

In terms of evidence generation, there is a need for more validation studies containing external test datasets and prospective datasets to demonstrate that automated tools are ready for downstream clinical requirements. When selecting patient cohorts, it is advisable to consider using consecutive or random samples, following a standard treatment protocol, avoiding inappropriate exclusions (ie, cherry-picking), and applying all necessary exclusions (eg, lack of histology, incomplete or poor-quality imaging data).

It is acknowledged that a focus on RANO assessment, especially for high-grade gliomas, fails to consider approaches other than fixed interval imaging where utility is unclear.^4,11^ It is also acknowledged that a focus on RANO assessment, especially for high-grade gliomas, and the use of T1C images alone in many studies, fails to use all the data produced during an MRI scan. Nonetheless, some studies included in the current review were not constrained to T1C only, potentially allowing for a more nuanced understanding of tumor behavior and response to treatment across different imaging and clinical scenarios. For example, in high-grade gliomas, DWI/ADC is a surrogate marker of tumor cell density and has been used to assess aggressiveness, while T2 contrast is effective in displaying broader tumor-host behavior.^56–59^ Future research may go beyond the limitations of RANO assessment and even incorporate advanced MRI as well as multi-modal techniques such as a combination of MRI and positron emission tomography.^60^

Similarly, treatment response assessment may be improved through another key area of research in neuro-oncology imaging which is the development of advanced MRI and radiomic prognostic, predictive, and monitoring biomarkers which have a role in treatment response assessment.^56^ Integrating radiomic biomarkers with automated longitudinal treatment response assessment frameworks offers a promising avenue for enhancing precision in tracking tumor responses across various brain tumor types.^54,61^

## Conclusion

The systematic review demonstrates the potential of automated tools to enhance the accuracy and reliability of treatment response assessments in brain tumors. Studies achieving complete automation from start to finish without manual intervention will contribute to the consistency and efficiency of data processing, likely minimizing the potential for human error and workload. However, automated tools are not clinic-ready and further research, especially incorporating external test datasets and prospective datasets, is now needed for more robust validation. Successful demonstration of tool use in the clinic or in clinical trials is also now required to complete clinical validation.

## Supplementary Material

noaf037_suppl_Supplementary_Table_S1

noaf037_suppl_Supplementary_Table_S2

noaf037_suppl_Supplementary_Table_S3

noaf037_suppl_Supplementary_Table_S4

## Data Availability

Data generated or analyzed during the study are available from the corresponding author by request.
