# Supplementary material for: Automated longitudinal treatment response assessment of brain tumors: A systematic review
Source: Neuro Oncol. 2025 Feb 12;27(8):1946–71. doi: 10.1093/neuonc/noaf037 (PMC12448867; doi:10.1093/neuonc/noaf037)
Supplement: noaf037_suppl_Supplementary_Table_S1 [file noaf037_suppl_supplementary_table_s1.docx]

**Supplementary Table S1.** PUBMED, EMBASE/OVID and Web of Sciences search strategies. (Different search terms used reflect the different constrains of the different databases)

PubMed

The search strategy for Title/Abstract terms used truncated search:

Database: PubMed <2010 to November 25, 2024>

Search Strategy:

--------------------------------------------------------------------------------

(‘Automated’ or ‘automatic’ or ‘pipeline’ or ‘AI’ or ‘artificial intelligence’ or ‘ML’ or ‘machine learning’ or ‘deep learning’ or ‘radiomics’) AND (‘brain tumor’ or 'brain metastases' or ‘glioma’ or ‘glioblastoma’) AND (‘longitudinal’ or ‘follow-up’ or ‘follow up’ or ‘treatment response’ or ‘monitoring biomarker’ or ‘response assessment’ or ‘monitoring’) AND (‘MRI’ or ‘magnetic resonance imaging’ or ‘magnetic resonance’ or ‘MR’) Filters: Chinese, English, from 2010/1/1 - 2024/11/25 (MeSH terms encompass both American and British spelling)

EMBASE (OVID).

Database: Embase <2010 to November 25, 2024 >

Search Strategy:

--------------------------------------------------------------------------------

1 exp glioblastoma/ or exp brain tumor/ or exp glioma/ (382216)

2 exp brain metastasis/ (48675)

3 exp radiomics/ (12931)

4 exp artificial intelligence/ (121509)

5 exp convolutional neural network/ or exp artificial neural network/ (121520)

6 exp machine learning/ (532247)

7 exp deep learning/ (68275)

8 MRI.mp. or exp nuclear magnetic resonance imaging/ (1409872)

9 automated.mp. (255009)

10 automatic.mp. (135931)

11 pipeline.mp. (59948)

12 exp longitudinal study/ (226724)

13 exp follow up/ (2282482)

14 exp treatment response/ (372048)

15 response assessment.mp. (11469)

16 exp monitoring/ (1122023)

17 1 or 2 (382216)

18 3 or 4 or 5 or 6 or 7 or 9 or 10 or 11 (933842)

19 12 or 13 or 14 or 15 or 16 (3764256)

20 8 and 17 and 18 and 19 (869)

21 limit 20 to yr="2010 - 2024" (851)

22 limit 22 to (chinese or english) (849)

23 limit 21 to (embase or medline) (611)

Web of Science

The search strategy for Title/Abstract terms used truncated search words:

Database: Web of Science < 2010 to November 25, 2024 >

Search Strategy:

--------------------------------------------------------------------------------

((((((((TS=(automated)) OR TS=(automatic)) OR TS=(pipeline)) OR TS=(artificial intelligence)) OR TS=(machine learning)) OR TS=(deep learning)) OR TS=(radiomics)) OR TS=(Neural Network)) AND ((((TS=(brain tumor)) OR TS=(brain metastasis)) OR TS=(glioblastoma)) OR TS=(glioma)) AND ((TS=(magnetic resonance imaging)) OR TS=(MRI) OR (TS=(magnetic resonance)) OR TS=(MR)) AND (((((((TS=(longitudinal)) OR TS=(follow up)) OR TS=(follow-up)) OR TS=(treatment response)) OR TS=(response assessment)) OR TS=(monitoring)) OR TS=(monitoring biomarker))

In the database search interface, the ‘Year’ filter was set to 2010-2024, and the ‘Language’ filter was applied to include only English and Chinese articles."
