# Supplementary material for: Automated longitudinal treatment response assessment of brain tumors: A systematic review
Source: Neuro Oncol. 2025 Feb 12;27(8):1946–71. doi: 10.1093/neuonc/noaf037 (PMC12448867; doi:10.1093/neuonc/noaf037)
Supplement: noaf037_suppl_Supplementary_Table_S2 [file noaf037_suppl_supplementary_table_s2.docx]

**Supplementary Table S2.** Tabular representation of the assessments assigned for each included study.

| **Study** | **Risk of bias** | | | | **Applicability** | | |
| --- | --- | --- | --- | --- | --- | --- | --- |
|  | Patient selection | Index test(s) | Reference standard | Flow and timing | Patient selection | Index test(s) | Reference standard |
| Chang, et al.^22^ | HIGH | HIGH | UNCLEAR | UNCLEAR | HIGH | HIGH | UNCLEAR |
| Nalepa, et al.^23^ | UNCLEAR | UNCLEAR | UNCLEAR | HIGH | UNCLEAR | UNCLEAR | UNCLEAR |
| Vollmuth, et al.^24^ | LOW | LOW | LOW | LOW | LOW | LOW | LOW |
| Rudie, et al.^25^ | LOW | HIGH | UNCLEAR | LOW | LOW | HIGH | UNCLEAR |
| Strack, et al.^26^ | HIGH | HIGH | HIGH | UNCLEAR | HIGH | HIGH | HIGH |
| Jalalifar, et al.^27^ | UNCLEAR | UNCLEAR | UNCLEAR | UNCLEAR | UNCLEAR | UNCLEAR | UNCLEAR |
| Kickingereder, et al.^28^ | HIGH | LOW | LOW | LOW | HIGH | LOW | LOW |
| Chen, et al.^29^ | UNCLEAR | UNCLEAR | UNCLEAR | UNCLEAR | UNCLEAR | UNCLEAR | UNCLEAR |
| Meier, et al.^30^ | HIGH | UNCLEAR | UNCLEAR | LOW | HIGH | UNCLEAR | UNCLEAR |
| Preetha, et al.^31^ | HIGH | LOW | LOW | LOW | HIGH | LOW | LOW |
| Cho, et al.^32^ | UNCLEAR | UNCLEAR | LOW | UNCLEAR | UNCLEAR | UNCLEAR | LOW |
| Hsu, et al.^33^ | UNCLEAR | UNCLEAR | UNCLEAR | LOW | UNCLEAR | UNCLEAR | UNCLEAR |
| Kleesiek, et al.^34^ | UNCLEAR | UNCLEAR | UNCLEAR | UNCLEAR | UNCLEAR | UNCLEAR | UNCLEAR |
| Ozkara, et al.^35^ | UNCLEAR | HIGH | HIGH | LOW | UNCLEAR | HIGH | HIGH |
| Suter, et.al 2023^36^ | UNCLEAR | HIGH | HIGH | UNCLEAR | UNCLEAR | HIGH | HIGH |
| Zhang, et al.^37^ | UNCLEAR | UNCLEAR | UNCLEAR | LOW | UNCLEAR | UNCLEAR | UNCLEAR |
| Prezelski, et al.^38^ | LOW | UNCLEAR | UNCLEAR | LOW | LOW | UNCLEAR | UNCLEAR |
| Son, et al.^39^ | UNCLEAR | HIGH | HIGH | LOW | UNCLEAR | HIGH | HIGH |
| Kotowski, et al.^40^ | UNCLEAR | HIGH | HIGH | LOW | UNCLEAR | HIGH | HIGH |
| Hammer, et al.^41^ | UNCLEAR | HIGH | HIGH | LOW | UNCLEAR | HIGH | HIGH |
