# Supplementary material for: Automated longitudinal treatment response assessment of brain tumors: A systematic review
Source: Neuro Oncol. 2025 Feb 12;27(8):1946–71. doi: 10.1093/neuonc/noaf037 (PMC12448867; doi:10.1093/neuonc/noaf037)
Supplement: noaf037_suppl_Supplementary_Table_S3 [file noaf037_suppl_supplementary_table_s3.docx]

**Supplementary Table S3.** Automated and semi-automated pipelines with region growing algorithm.

| **Study** | **Dataset**  **1. Tumor type**  **2. Retrospective/Prospective**  **3. Single/Multiple sites**  **4. Sequences**  **5. Numbers** | **Pipeline steps** | **Automated model(s) (index test(s), in comparison with reference standard(s)** | **Performance metrics for tasks prior to longitudinal assessment e.g., segmentation** | **Longitudinal performance metrics** |
| --- | --- | --- | --- | --- | --- |
| Huber, et al.^51^ | Dataset 1 longitudinal local data:  1. Glioblastoma (pre 2021 WHO classification)  2. Retrospective  3. Single-site  4. T1, FLAIR  5. 30 patients with 330 MRIs | 1. Registration  2. Segmentation  3. Calculations of regional, segmentation-based subtractions and absolute change of tumor volumes. | Semi-automated software Smartbrush by region growing algorithm compared to neuro-radiologic expertise for segmentation.  Automated compared to manual volume change assessment. | N/A | a. ROC-curves of the prediction of disease progression (Dataset 1)  delta (absolute volume) AUC = 0.83; Sub (regional volume subtractions) AUC = 0.75  b. Accuracy AUC of GLMM (Dataset 1)  delta AUC (95% CI) = 0.79 (0.70 ± 0.86); sub-AUC (95% CI) = 0.73 (0.63 ± 0.81) |
| Bauknecht, et al.^52^ | Dataset 1 Longitudinal Dataset:  1. Brain metastases  2. Retrospective  3. Single-site  4. T1 C  5. 38 patients with 262 MRIs (131 baseline and 131 follow-up) | 1. Segmentation  2. Largest diameter generation  3. Classification of treatment  response | Index test is software OncoTREAT using an algorithm of region growing and morphologic criteria compared to Reference standard is manual measurement for segmentation and diameters generation and classification by RANO-BM^7^. | N/A | a. Correlation coefficients for intra-observer variability (single observer at different timepoints) = 0.993 (Dataset 1)  b. The interobserver variability (R1/R2) (consistency measured by different observers) = (0.989/0.998) (Dataset 1)  c. Accuracy of classification = 75% (Dataset 1) |
| Tan, et al.^53^ | Dataset 1 Longitudinal Dataset:  1. Glioma and brain metastasis  2. Retrospective  3. Single-site  4. T1 C  5. 20 patients with 58 MRIs | 1. Segmentation  2. Size change calculation  3. Treatment response Classification | Multimodal Tumor Tracking software (based on regional growth and morphological image processing algorithms) compared to manual measurement based on RECIST 1.1 (total volume and max 3D diameter change). | N/A | a. ROC Curve performance (Dataset 1)  AUC (Total Volume, PR Threshold) = 1.00; AUC (Total Volume, PD Threshold) = 0.95  AUC (Max 3D Diameter, PR Threshold) = 0.86; AUC (Max 3D Diameter, PD Threshold) = 0.97 |
