# Supplementary material for: Automated longitudinal treatment response assessment of brain tumors: A systematic review
Source: Neuro Oncol. 2025 Feb 12;27(8):1946–71. doi: 10.1093/neuonc/noaf037 (PMC12448867; doi:10.1093/neuonc/noaf037)
Supplement: noaf037_suppl_Supplementary_Table_S4 [file noaf037_suppl_supplementary_table_s4.docx]

**Supplementary Table S4.** QUADAS-2 Bias Risk Assessment for Each Article.

| **Domains** | **SStudy**  **Assessment Criteria** | Chang, et al.^22^ | Nalepa, et al.^23^ | Vollmuth, et al.^24^ | Rudie, et al.^25^ | Strack, et al.^26^ | Jalalifar, et al.^27^ | Kickingereder, et al.^28^ | Chen, et al.^29^ | Meier, et al.^30^ | Preetha, et al.^31^ | Cho, et al.^32^ | Hsu, et al.^33^ | Kleesiek, et al.^34^ | Ozkara, et al.^35^ | Suter, et al. 2023^36^ | Zhang, et al.^37^ | Prezelski, et al.^38^ | Son, et al.^39^ | Kotowski, et al.^40^ | Hammer, et al.^41^ |
| --- | --- | --- | --- | --- | --- | --- | --- | --- | --- | --- | --- | --- | --- | --- | --- | --- | --- | --- | --- | --- | --- |
| Patient selection | Was a consecutive or random sample of patients enrolled? | NO | UNCLEAR | YES | YES | NO | UNCLEAR | NO | UNCLEAR | YES | NO | YES | YES | UNCLEAR | YES | UNCLEAR | YES | YES | YES | UNCLEAR | UNCLEAR |
|  | Was a case-control design avoided? | YES | YES | YES | YES | YES | YES | YES | YES | YES | YES | YES | UNCLEAR | YES | YES | YES | YES | YES | YES | YES | YES |
|  | Was a standard treatment protocol or guideline followed (e.g. Stupp protocol for glioblastoma)? | YES | UNCLEAR | YES | YES | UNCLEAR | YES | UNCLEAR | UNCLEAR | YES | UNCLEAR | UNCLEAR | UNCLEAR | YES | YES | YES | UNCLEAR | YES | UNCLEAR | UNCLEAR | YES |
|  | Did the study avoid inappropriate exclusions (i.e. cherry picking)? | YES | UNCLEAR | YES | YES | YES | UNCLEAR | YES | UNCLEAR | NO | YES | UNCLEAR | YES | UNCLEAR | YES | UNCLEAR | UNCLEAR | YES | YES | UNCLEAR | YES |
|  | Did the study apply all appropriate exclusions (e.g. no histology, incomplete or poor-quality imaging data)? | YES | UNCLEAR | YES | YES | UNCLEAR | UNCLEAR | YES | YES | UNCLEAR | YES | YES | UNCLEAR | UNCLEAR | UNCLEAR | YES | YES | YES | YES | UNCLEAR | YES |
|  | Could the selection of patients have introduced bias? (Risk of bias) | HIGH | UNCLEAR | LOW | LOW | HIGH | UNCLEAR | HIGH | UNCLEAR | HIGH | HIGH | UNCLEAR | UNCLEAR | UNCLEAR | UNCLEAR | UNCLEAR | UNCLEAR | LOW | UNCLEAR | UNCLEAR | UNCLEAR |
|  | Concerns regarding applicability. Is there concern that the included patients do not match the review question? (Concerns regarding applicability) | HIGH | UNCLEAR | LOW | LOW | HIGH | UNCLEAR | HIGH | UNCLEAR | HIGH | HIGH | UNCLEAR | UNCLEAR | UNCLEAR | UNCLEAR | UNCLEAR | UNCLEAR | LOW | UNCLEAR | UNCLEAR | UNCLEAR |
| Index test(s) | Were the index test results interpreted without knowledge of the results of the reference standard (i.e. no overlap between training and test sets)? | YES | UNCLEAR | YES | YES | YES | UNCLEAR | YES | YES | YES | YES | YES | YES | YES | YES | YES | YES | YES | YES | YES | YES |
|  | If a threshold was used, was it pre-specified (i.e. after training, the model parameters were fixed and not changed to optimise the test set)? | YES | YES | YES | YES | YES | YES | YES | UNCLEAR | YES | YES | YES | YES | YES | YES | YES | YES | YES | YES | UNCLEAR | UNCLEAR |
|  | Does the interpretation of the reference standard results account for relevant epidemiological, clinical and molecular characteristics (e.g. age, performance status, steroids, MGMT methylation status, 2nd- and 3rd-line chemotherapies, antiangiogenics, immunotherapies, reoperation)? | NO | UNCLEAR | YES | NO | NO | UNCLEAR | YES | UNCLEAR | UNCLEAR | YES | UNCLEAR | UNCLEAR | UNCLEAR | NO | NO | UNCLEAR | UNCLEAR | NO | NO | NO |
|  | Could the conduct or interpretation of the index test have introduced bias? (Risk of Bias) | HIGH | UNCLEAR | LOW | HIGH | HIGH | UNCLEAR | LOW | UNCLEAR | UNCLEAR | LOW | UNCLEAR | UNCLEAR | UNCLEAR | HIGH | HIGH | UNCLEAR | UNCLEAR | HIGH | HIGH | HIGH |
|  | It is unclear whether the index test results were interpreted in the context of any confounding clinical information. Is there concern that the index test, its conduct or interpretation differ from the review question? (Concerns regarding applicability) | HIGH | UNCLEAR | LOW | HIGH | HIGH | UNCLEAR | LOW | UNCLEAR | UNCLEAR | LOW | UNCLEAR | UNCLEAR | UNCLEAR | HIGH | HIGH | UNCLEAR | UNCLEAR | HIGH | HIGH | HIGH |
| Reference standard | Is the reference standard likely to correctly classify the target condition? | YES | YES | YES | YES | YES | YES | YES | YES | YES | YES | YES | YES | YES | YES | YES | YES | YES | YES | NO | YES |
|  | Were the reference standard results interpreted without knowledge of the results of the index test? | YES | YES | YES | YES | YES | UNCLEAR | YES | UNCLEAR | UNCLEAR | YES | YES | UNCLEAR | YES | YES | YES | YES | YES | YES | YES | YES |
|  | Does the interpretation of the reference standard results account for relevant epidemiological, clinical and molecular characteristics (e.g. age, performance status, steroids, MGMT methylation status, 2nd- and 3rd-line chemotherapies, antiangiogenics, immunotherapies, reoperation)? | UNCLEAR | UNCLEAR | YES | UNCLEAR | NO | UNCLEAR | YES | UNCLEAR | UNCLEAR | YES | YES | UNCLEAR | UNCLEAR | NO | NO | UNCLEAR | UNCLEAR | NO | NO | NO |
|  | Could the reference standard, its conduct, or its interpretation have introduced bias? (Risk of Bias) | UNCLEAR | UNCLEAR | LOW | UNCLEAR | HIGH | UNCLEAR | LOW | UNCLEAR | UNCLEAR | LOW | LOW | UNCLEAR | UNCLEAR | HIGH | HIGH | UNCLEAR | UNCLEAR | HIGH | HIGH | HIGH |
|  | Is there concern that the index test, its conduct or interpretation differ from the review question? (Concerns regarding applicability) | UNCLEAR | UNCLEAR | LOW | UNCLEAR | HIGH | UNCLEAR | LOW | UNCLEAR | UNCLEAR | LOW | LOW | UNCLEAR | UNCLEAR | HIGH | HIGH | UNCLEAR | UNCLEAR | HIGH | HIGH | HIGH |
| Flow and timing | Was there an appropriate interval between index test(s) and reference standard? | UNCLEAR | UNCLEAR | YES | YES | UNCLEAR | UNCLEAR | YES | UNCLEAR | YES | YES | UNCLEAR | YES | UNCLEAR | YES | UNCLEAR | UNCLEAR | UNCLEAR | YES | YES | YES |
|  | Did all patients receive a reference standard? | YES | UNCLEAR | YES | YES | YES | YES | YES | YES | YES | YES | YES | YES | YES | YES | YES | YES | YES | YES | YES | YES |
|  | Did patients receive the same reference standard? | YES | YES | YES | YES | YES | YES | YES | YES | YES | YES | YES | YES | YES | YES | YES | YES | YES | YES | YES | YES |
|  | Were all patients included in the analysis? | YES | NO | YES | YES | YES | YES | YES | YES | YES | YES | YES | YES | UNCLEAR | YES | YES | YES | YES | YES | YES | YES |
|  | Could the patient flow have introduced bias? (Risk of Bias) | UNCLEAR | HIGH | LOW | LOW | UNCLEAR | UNCLEAR | LOW | UNCLEAR | LOW | LOW | UNCLEAR | LOW | UNCLEAR | LOW | UNCLEAR | LOW | LOW | LOW | LOW | LOW |
